# Single Vesicle Surface Protein Profiling and Machine Learning-Based Dual Image Analysis for Breast Cancer Detection

**DOI:** 10.3390/nano14211739

**Published:** 2024-10-30

**Authors:** Mitchell Lee Taylor, Madhusudhan Alle, Raymond Wilson, Alberto Rodriguez-Nieves, Mitchell A. Lutey, William F. Slavney, Jacob Stewart, Hiyab Williams, Kristopher Amrhein, Hongmei Zhang, Yongmei Wang, Thang Ba Hoang, Xiaohua Huang

**Affiliations:** 1 Department of Chemistry, The University of Memphis, Memphis, TN 38152, USA; mltylor3@memphis.edu (M.L.T.); mralle@memphis.edu (M.A.); rwlson14@memphis.edu (R.W.J.); lrdrgez6@memphis.edu (A.R.-N.); malutey@memphis.edu (M.A.L.); wfslvney@memphis.edu (W.F.S.); kristopher-amrhein@utc.edu (K.A.); ywang@memphis.edu (Y.W.); 2 Department of Physics and Materials Science, The University of Memphis, Memphis, TN 38152, USA; j.stewart@memphis.edu (J.S.); hjwllams@memphis.edu (H.W.); tbhoang@memphis.edu (T.B.H.); 3 School of Public Health, The University of Memphis, Memphis, TN 38152, USA; hzhang6@memphis.edu

**Keywords:** machine learning, single-vesicle technology, breast cancer, extracellular vesicle, gold nanoparticles, optical imaging

## Abstract

Single-vesicle molecular profiling of cancer-associated extracellular vesicles (EVs) is increasingly being recognized as a powerful tool for cancer detection and monitoring. Mask and target dual imaging is a facile method to quantify the fraction of the molecularly targeted population of EVs in biofluids at the single-vesicle level. However, accurate and efficient dual imaging vesicle analysis has been challenging due to the interference of false signals on the mask images and the need to analyze a large number of images in clinical samples. In this work, we report a fully automatic dual imaging analysis method based on machine learning and use it with dual imaging single-vesicle technology (DISVT) to detect breast cancer at different stages. The convolutional neural network Resnet34 was used along with transfer learning to produce a suitable machine learning model that could accurately identify areas of interest in experimental data. A combination of experimental and synthetic data were used to train the model. Using DISVT and our machine learning-assisted image analysis platform, we determined the fractions of EpCAM-positive EVs and CD24-positive EVs over captured plasma EVs with CD81 marker in the blood plasma of pilot HER2-positive breast cancer patients and compared to those from healthy donors. The amount of both EpCAM-positive and CD24-positive EVs was found negligible for both healthy donors and Stage I patients. The amount of EpCAM-positive EVs (also CD81-positive) increased from 18% to 29% as the cancer progressed from Stage II to III. No significant increase was found with further progression to Stage IV. A similar trend was found for the CD24-positive EVs. Statistical analysis showed that both EpCAM and CD24 markers can detect HER2-positive breast cancer at Stages II, III, or IV. They can also differentiate individual cancer stages except those between Stage III and Stage IV. Due to the simplicity, high sensitivity, and high efficiency, the DISVT with the AI-assisted dual imaging analysis can be widely used for both basic research and clinical applications to quantitatively characterize molecularly targeted EV subtypes in biofluids.

## 1. Introduction

The ability to molecularly diagnose cancer at different stages has a high impact on clinical care, including diagnosing early cancer, identifying cancer subtypes, predicting prognosis, directing treatment, and monitoring treatment response and cancer remission. Particularly, early detection remains the key to improving survival. For example, the overall 5-year survival rate for women diagnosed with breast cancer at Stage I is 99%, while those women diagnosed with Stage IV breast cancer only have about a 29% chance of survival [1]. But only 61% of breast cancers are diagnosed at early stages (Stages I and II) using existing techniques [2]. It is, therefore, clear that there is a need for better methods that can detect early cancer and different stages.

Extracellular vesicles (EVs) are a heterogeneous group of cell-derived membrane-bound vesicles comprising exosomes and microvesicles [3]. They are continually released by nearly all cells. The DNA, RNA, and various proteins that comprise EVs mirror those of their parental cells [4,5,6]. EVs have been implicated as important mediators of intercellular communication, whereby they promote many pathophysiological disorders, including cancer, neurodegeneration, and inflammatory disease [7,8,9,10,11]. Tumor-derived exosomes enter blood and many other body fluids such as urine, saliva, ascites, and cerebrospinal fluid [12,13,14,15,16]. The concentration of EVs in the blood has been reported to be between 0.88 × 10^8^ and 13.14 × 10^8^ exosomes/mL [17], which is a noteworthy concentration when considering diagnostic potential. Exosomes are fairly stable, tolerating multiple cycles of freezing and thawing, and can be preserved for years when stored in liquid nitrogen (LN) [18,19]. Thus, EVs hold strong promises for the development of next-generation liquid biopsy for cancer as well as a plethora of other diseases [20,21,22,23,24].

Liquid biopsies are particularly desired as diagnostic tools over many other options, such as surgical biopsies, due to their non-invasive nature, ease of application, and accuracy [25]. However, while EVs are effective targets in liquid biopsies, some issues remain regarding their usefulness. One major issue currently being researched is the fact that Evs are heterogeneous—there are many exosomes in the blood and other bodily fluids originating from many different cell types, including diseased cells and healthy cells, and these populations are difficult to separate during analysis [26]. For this reason, the typical liquid biopsies using EVs relying on ‘bulk’ analysis of their populations, such as Western Blot or Enzyme-Linked Immunosorbent Assay (ELISA), cannot reliably detect and categorize subpopulations of exosomes [27]. Therefore, it is likely that a signal from a subpopulation will be buried in the ensemble average signal of the dominating EV populations [28]. For this reason, many researchers are now investigating the detection of single vesicles to more accurately describe the EV environment within a sample [27,29,30,31,32].

Single EV detection is typically broken up into two main groups: label-free methods and label-based methods. Label-free methods include Nanoparticle Tracking Analysis (NTA), Raman Tweezers [33], Transmission and Scanning Electron Microscopy, Cryo-EM, and Atomic Force Microscopy (AFM) [34,35,36], while label-based methods include fluorescence detection [37,38], high-resolution flow cell cytometry [39], total internal reflection microscopy [40], FRET [41], and super-resolution microscopy [28].

We recently developed a single vesicle detection approach based on label-based, dual fluorescent, and darkfield imaging [24]. We dubbed this method Dual Imaging Single-Vesicle Technology (DISVT). Briefly, for this experiment, we created a dual imaging mode apparatus where fluorescence tags are embedded in the membrane of EVs present in a microliter-sized sample, while gold nanoparticles (AuNPs) are conjugated to target-specific antibodies such as anti-HER2 antibodies to detect surface cancer markers. Only those EVs present in the sample that express the targeted cancer-related protein should therefore bind the conjugated AuNPs. The membrane-bound fluorescent tags are detected by laser excitation, which counts the total number of exosomes present in each image. By utilizing darkfield microscopy, we obtain images of the subpopulation of those same exosomes that have bound AuNPs, thus yielding the fraction of EVs that are positive to the target protein markers relative to the total number of exosomes imaged in the fluoresce mode. In this experiment, we were able to demonstrate a significant difference between early-stage breast cancer (Stages I and II) and stage III breast cancer based on HER2 expression on plasma EVs.

In this work, we examine the potential of EVs to differentiate all four stages of breast cancer with a proof-of-concept study. We chose cancer markers CD24 and EpCAM due to their association with breast cancer. CD24, which is a regulator of cell migration, invasion, and proliferation in certain cancers, is often overexpressed in tumors and is a potential biomarker for breast cancers, as CD24 overexpression is associated with poor prognosis [42]. EpCAM has been known to be overexpressed in certain breast cancers for almost two decades and is also a marker for poor prognosis [43]. However, it is not clear how their expressions on EVs are associated with breast cancer, particularly HER2-positive breast cancer that already has HER2 for the diagnosis of this type of cancer.

One major hurdle during the analysis of the hundreds of produced images with DISVT is being able to efficiently and accurately count an acceptable fraction of the total EVs present in each image. In our previous work, we used a combination of ImageJ, Bash, and Python (scripted through a Python wrapper in ImageJ) to count the EVs as quickly as possible. This method works well in many cases but is technically difficult to carry out for anyone unfamiliar with the analysis process. It is also slower than a fully automated method. Furthermore, the potential commercialization of the experiment is prohibitory unless a fast and automated image analysis pipeline easily carried out by a non-expert can be developed. For these reasons, we have developed a machine learning image analysis platform using transfer learning that can identify areas of interest quickly and effectively.

Applying machine learning to image data to identify cancer and other diseases is not a new approach for medical imaging purposes, and many groups have already applied this technique. For example, Khan et al. applied transfer learning for the detection and classification of breast cancer using cytology images [44]. They compared multiple deep learning models and found their approach outperformed many other deep learning approaches in terms of accuracy. Bello et al. used a modified version of Densenet to increase the accuracy of the current machine learning approach at that time to identify skin cancers in image data to 87% [45]. Their method demonstrated the importance of using a fine-tuned neural network for cancer classification when using images. Ram et al., reviewed AI applications to the diagnosis, treatment, and prognosis of chronic myeloid leukemia, where they found that most machine learning models applied to this area are related to diagnosis and prediction [46]. Of the models surveyed, it was found convolutional neural networks were a common approach to assist in diagnosis and prediction.

In this work, we developed a fully automated AI-based method that can simultaneously analyze multiple pairs of mask/target images. Using the machine learning-based imaging analysis and the DISVT, we quantified EVs that are positive for EpCAM or CD24 and examined their ability to detect HER2-positive breast cancer at different stages. We found that the amount of the EpCAM-positive EVs (also CD81-positive) or the CD24-positive EVs increased as the cancer progressed from Stage II to III. No significant increase was found with further progression to Stage IV. Statistical analysis revealed that both markers can detect HER2-positive breast cancer at Stage II, III, and IV and differentiate different stages except Stage III and IV. The DISVT with the machine learning-assisted dual imaging analysis can be widely used for both basic research and clinical applications to quantitatively characterize molecularly targeted EV subtypes in biofluids.

## 2. Materials and Methods

### 2.1. Materials

All reagents were purchased from Millipore Sigma (St. Louis, MO, USA) unless otherwise specified. Antibodies were purchased from Biolegend (San Diego, CA, USA). N-hydroxysuccinimide-poly(ethylene glycol)-thiol (NHS-PEG-SH, MW1000), methoxy-PEG-SH (MW5000), and cholesterol-PEG-Cy5 (Chol-PEG-Cy5, MW2000) were purchased from Nanocs (Boston, MA, USA).

### 2.2. Source of EVs from Patients and Healthy Donors

Human plasma samples from healthy donors and breast cancer patients were obtained from BioIVT, Inc. (Westbury, NY, USA). The plasma was diluted with phosphate-buffered solution (PBS) 20−1000 times depending on the subjects (final concentration of ~109 EVs/mL) and filtered with a 0.2 μm polyethersulfone (PES) filter (Agilent Technologies, Santa Clara, CA, USA) before use.

### 2.3. Single EV Detection with Dual Imaging Single-Vesicle Technology

Targeted surface protein markers on individual EVs were detected using the dual imaging single-vesicle technology (DISVT) that we recently developed [24]. Briefly, ultra-flat Au film (100 nm in thickness) on a standard microscopic glass slide was prepared by a template stripping method using a commercial Au-coated wafer (Angstrom Engineering Inc., Cambridge, ON, Canada). The Au chamber slide was separated into multiple wells (5 mm in diameter) with black vinyl tape to form an Au chamber slide. The wells were coated with anti-CD81 rabbit monoclonal antibodies linked with NHS-PEG-SH 1000 and then saturated with 11-mercaptoundecyl tetra (ethylene glycol) (MUTEG).

EVs were captured onto the Au chamber slide by incubating diluted and filtered plasma samples in the functionalized wells for 2 h at room temperature (RT). After washing with PBS containing 0.01% tween 20 (PBST0.01%), the EVs were incubated with antibody-conjugated AuNPs (50 pM in PBST0.05%) for 1 h at RT. The antibody-conjugated AuNPs were prepared by linking target-specific antibodies to Au NPs (60 nm in diameter, Ted Pella, Inc., Redding, CA, USA) via NHS-PEG-SH 1000. At last, the EVs were incubated with 20 µM of Chol-PEG-Cy5 (15 min, 37°C) to label the lipid membrane. After washing with PBS, the EVs were quickly rinsed with water, dried with nitrogen, and imaged immediately.

Dark field and fluorescence images were acquired with a dual imaging system that was constructed on a customized Nikon LV150N microscope (Melville, NY, USA) with a 3D nanometer resolution translation stage (model 9063, Newport, Irvine, CA, USA). The dark field images were illuminated with a halogen lamp. The fluorescence images were accomplished with a Melles Griot continuous-wave He laser (model 05-LPH-925, l = 632.8 nm) in an angled direction (∼45°, relative to the sample surface). All images were collected through an extra-long working distance (4.5 mm), high numerical aperture (NA = 0.8), 100× Bright/Dark field objective lens by a photometrics CoolSNAP camera (Teledyne Photometrics, Tucson, AZ, USA).

### 2.4. Machine Learning Network Architecture

The network architecture was built using the Segmentation Models library based on TensorFlow and Keras. The convolutional neural network (CNN) Resnet34, which had been pre-trained on the 2012 ILSVRC ImageNet dataset, was used as the backbone of our UNET machine learning model. The model resembles a typical CNN used for image segmentation purposes, consisting of a contracting path and an expansive path. The contracting path is used to extract useful features from the image, while the expansive path combines the extracted features together and then feeds the result into a SoftMax classifier. The input image is either an experimentally gathered image or a synthetically generated image, and the output is each image’s segmentation map, which is the same size as that of the input image.

The contracting path of Resnet34 consists of repeated applications of 3 × 3 downsampling convolutions, each one followed by a ReLU and a batch normalization. Skip connections are used to combat the vanishing gradient problem. The expansive path uses a series of convolutions to map each feature vector to the number of classes and deconvolutions and concatenations to build the resulting image to the same size as the input image. Batch normalizations are used throughout. The exact model can be found in the Segmentation Models library.

There are many machine learning approaches and models that are used for image segmentation purposes; however, we chose to use the pre-trained Resnet34 model because it yielded fast and accurate results. There are certainly other models in the Segmentation Models library that could offer similar results, but we found Resnet34 to be sufficient for our needs. Furthermore, the pre-trained model decreased the training time tremendously, as an untrained model would have required much more time and computing resources.

### 2.5. Auto Single EV Dual Imaging Analysis (AutoSEDIA)

A custom Python image segmentation software was developed using standard algorithmic approaches to rapidly create segmentation maps for most of our experimental image data. The software is near automatic, yet still requires some minor user input due to variability in some experimental parameters, such as background signal. The first step in the software pipeline is to take an estimation of the background of the raw experimental image. This step helps eliminate extraneous reflected and scattered light from the surface of the sample as well as from the sample itself. The estimated background was found by first smoothing the image by applying a zeroth order Savitzky–Golay smoothing filter of window size 3, then applying a reciprocal Fourier Gaussian filter to the reciprocal of the raw image. The Fourier Gaussian function from the SciPy library was used for this purpose. Any pixel intensities that remained higher in the filtered image were replaced with the lower values of the original smoothed image. This process was repeated for a total of 10 iterations and produced 8-bit images. These 8-bit images were saved and used to create the training dataset and would henceforth be referred to as the experimental training dataset. Next, a signal-to-background image was produced by dividing the raw image by the estimated background. Blob detection was accomplished by applying the Fourier Gaussian of the resulting SBR image, followed by the Laplacian of the resulting image, where the Laplacian function was taken from the SciPy library. The resulting image was an eight-bit, greyscale image where higher values constituted features and lower values were the background. The adaptive thresholding function from the CV library was then used to locally determine each feature from the background; the resulting image was converted into a binary image, and then the function of the clear border from the sci-kit image was used to ensure only features not interacting with the border of the image remain in the feature space. An image closing was carried out by using the area closing function from the sci-kit image, followed by a custom vectorized hole-filling function to fill all holes in each feature. A watershed was carried out via the watershed function from the sci-kit image. Small objects were removed using the remove small objects function from the morphology library from the sci-kit image, and large objects were removed using a modified version of that same function. The resulting image was then the final segmentation mask.

### 2.6. Experimentally Gathered Training Data

Experimentally produced images generated from targeting EpCAM, CD24, or HER2 (the HER2 protein was the protein of interest targeted in past experiments detailed in our previous paper, which was mentioned in the introduction of this paper) were used to create a training dataset. These images were gathered as 16-bit, 1920 × 1460, grayscale and consisted of a total number of 100 images, 50 of them target and 50 of them mask images. To produce segmentation masks, either ImageJ or AutoSEDIA is used. Both methods produce practically identical results. ImageJ is used only in those cases where AutoSEDIA fails to produce correct segmentation masks, which are determined upon visual inspection.

AutoSEDIA is first used to create the 8-bit experimental training dataset as described in the previous section. If AutoSEDIA fails to produce an accurate segmentation map for a given image set, then ImageJ was used instead. The ImageJ pipeline consists of taking the 8-bit filtered images produced from AutoSEDIA and setting any intensities that were lower than a value of 3 to 0, designating a background. Then the native smoothing filter from ImageJ is applied as many times as required, and then the threshold is set manually and adjusted as needed. The result is inspected visually. Finally, the image is converted to binary and inverted to produce black (0 s) for the background and white (1 s) for the foreground, yielding the final segmentation mask.

The resulting 8-bit images produced from AutoSEDIA and their corresponding segmentation masks produced by either AutoSEDIA or ImageJ are then cropped into smaller 256 × 256 patches. Only the patches containing at least 5% of pixel values of value 10 or greater, these pixels being regions of interest and not background, are kept for training. A total of 847 of 256 × 256 patched training images were produced.

### 2.7. Synthetic Training Data Generation

Additional CNN training data were generated using a variational auto-encoder (VAE) machine learning model. The model architecture consists of an encoder and decoder scheme. For the encoder, two convolutions are applied in which ReLU activations follow each convolution function, followed finally by a final dense layer. For the decoder, a dense layer is applied, followed by a ReLU activation function, and then two transpose convolutions are applied, each followed by a ReLU activation function. Lastly, a final transpose convolution is applied with a sigmoid activation function.

The VAE training data consisted of 50 experimentally gathered data in an equal amount of mask and target images of size 1920 × 1460. These 8-bit experimentally gathered images from either EpCAM, CD24, or HER targeting where the filtered images were generated from AutoSEDIA. These images were then patched into 28 × 28 size patches, and only those patches that contained at least 5% useful information (pixel values of 10 or greater) were kept for training. The model then was able to produce as many 28 × 28 synthetically generated patches as necessary. To create the 256 × 256 patch sizes needed for CNN training, randomly selected 28 × 28 synthetically generated images were stitched together to form 256 × 256 sized images for CNN training.

Two methods were used to validate the generated synthetic data and ensure they were representative of the original experimental images. The first method was visualization and numerical inspection of a random sample of 50 of the generated images. The morphology and density of the produced features were checked visually, while the sizes and intensities were checked numerically. Visualization remained a useful method to ascertain the training performance because both the training and produced images were small, 28 × 28 8-bit patches and contained little information, making visual inspection fast and easy. The second method uses only synthetic data to train the CNN model and then uses that model to predict the experimental images. Adam was used for optimization with a learning rate of 0.001 with a batch size of 25. Training the model for 50 epochs produced images most closely resembling the training dataset.

### 2.8. CNN Network Training

A pixel-wise SoftMax classifier was used to predict the class labels with a Categorical Jaccard loss function where the loss is summed from the batches. The Adam algorithm was chosen for optimization. The weights were initialized using the weights from the Resnet model, which had been pre-trained on the ImageNet database, which contained almost 400,000 training images. A batch size of 32 was used, and the model was trained for 50 epochs. The training took approximately 45 min on a single 16 GB NVIDIA 3080 TI GPU (Nvidia Inc., Santa Clara, CA, USA).

### 2.9. Data Analysis

After the regions of interest (ROI) in the images were segmented using CNN, a custom Python script utilizing the Python library scikit-image was used to label each ROI in both the target and mask segmentation map images. The locations of the ROIs in each image were superimposed, and those in which the target and mask ROI areas coincided with one another were recorded as marker-positive EVs. The integrated average SBR pixel intensities in the unit area of the labeled ROIs in the dark-field image at the EV mask locations gave the population density histogram that included two populations, AuNP-bound EVs and AuNP-free EVs. The fraction of marker-positive EVS to the total EV population, F_p_, was then calculated by taking the difference between the fraction of Au-bound EVs using the antibody-conjugated AuNPs and the fraction of Au-bound EVs using the IgG-conjugated AuNPs. The total F_p_ was found for every image in an image set, where an image set consists of 8 images and the values were then averaged to yield a total average F_p_.

### 2.10. Model Performance Evaluation

The CNN model was tested on several synthetic and experimentally produced images that had been meticulously labeled and reviewed for accuracy and on which the model had not been trained. All images were randomly selected from a test image set consisting of 50 images, evenly distributed between synthetic and experimental 1920 × 1460 sized images. Background estimation and removal were conducted in the same manner as the training images, then the images were patched into 256 × 256 segments. The CNN model was then used to predict the labels, and each complete 1920 × 1460 full-size segmentation map was constructed by stitching together each 256 × 256 patch that had gone through prediction.

Mean intersection over union (IOU) was used for pixel-level semantic segmentation evaluation to determine the model’s effectiveness. Let *n_ij_* be the number of pixels of class *i* predicted to belong to class *j*, where *k* represents the different classes. Then, mean IOU is defined as follows:

$$\frac{1}{k}\sum_{i}\frac{n_{ii}}{\sum_{j}n_{ij}+\sum_{j}n_{ji}-n_{ii}}$$

## 3. Results and Discussions

### 3.1. AutoSEDIA

There are numerous standard algorithmic approaches that have been developed for image segmentation needs, and it is possible to use these approaches in an automated fashion. These methods have been researched and used for many years and are usually robust; however, there are cases, such as in our experiments, where the standard algorithms fail to be applied usefully because of some variability in the experimental parameters. For example, in our single-vesicle experiments, which are highly sensitive because of the high magnification, small changes in the environment introduce significant negative effects that complicate the automated image analysis process. These small changes may be aggregations from the fluorescent tags or variations in background signal, either of which will cause the highly tuned algorithmic pipeline to fail. This is one reason why machine learning has been so effective for many image segmentation tasks. It is much easier to let the algorithm decide what information is relevant rather than attempting to continually correct the complicated standard algorithmic pipeline. Additionally, machine learning offers the ability to update the model with new data should new information present itself, which is relevant for the success of the segmentation process. However, isolating regions of interest from experimental image data and creating their respective segmentation maps for the training images, which is required for machine learning to work, is a time-consuming process; therefore, it is useful to have some automation when creating the training data if possible. We created AutoSEDIA for this purpose, which can process more than 50% of all our image data. For the remaining images, which may pose problems for AutoSEDIA, we use ImageJ instead. While much slower than AutoSEDIA, which can process an image pair in less than 5 s, it is more versatile since the open-source program contains a plethora of image analysis algorithms, all of which are applied manually and visually. Therefore, the usefulness of both methods culminated in the final automated machine learning model. Of the 100 experimental images, 33 of them were prepared for training using ImageJ, while the remaining 67 were prepared using the image analysis algorithms of AutoSEDIA. The average fraction of EVs per image was 643.

### 3.2. The Training Data

**Figure 1:**
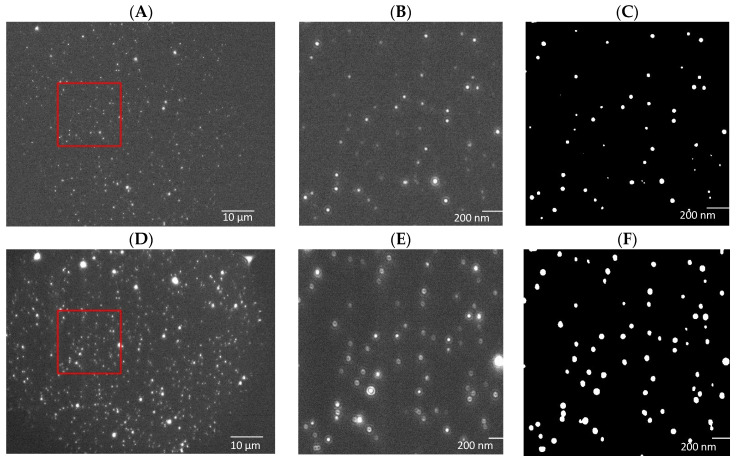
Original (1920 × 1460) florescence mask images (**A**) and dark field target images (**D**) each with a 256 × 256 patch example (**B**,**E**) and their associated segmentation map (**C**,**F**). (**B**,**C**) images are the zoom in region in the red square of image (**A**). (**E**,**F**) images are the zoom in region in the red square of image (**D**). Images were acquired using plasma EVs from a Stage III patient with EpCAM as the targeted surface marker.

The training dataset of images was half experimentally gathered data and half synthetically generated data generated using a variational auto-encoder machine learning model trained on experimental data. An example of a mask and target image pair from the EpCAM experimental dataset can be seen in Figure 1. Here, the full-size 1920 × 1460 16-bit images can be seen, as can an example of a 256 × 256 8-bit patch for each image and their corresponding segmentation masks. The reason patching was used was that sufficient batch sizes were not possible given the limited amount of GPU memory; therefore, the images were first patched into smaller images, and then the segmentation masks were later reconstructed to full size by stitching. This process is not especially complicated and is simply a matter of keeping track of which patch is being predicted and what pixel values and their locations constitute each patch from the original 1920 × 1460 8-bit image. The total number of images either gathered experimentally or generated is shown in Table S1.

**Figure 2:**
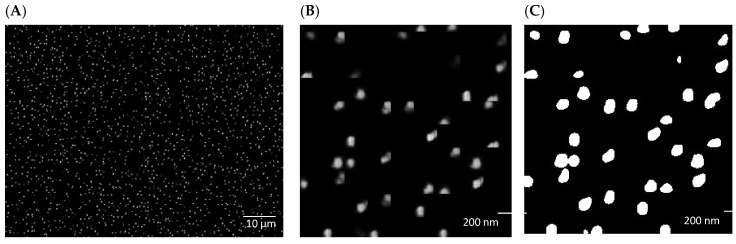
Synthetic image example (**A**) with 256 × 256 patch (**B**) and its associated segmentation map (**C**).

AutoSEDIA was able to produce the needed 8-bit images regardless of the automation failing to produce an accurate segmentation mask. This is because the 8-bit image will contain much noise around each ROI, but the CNN is able to learn which pixels are noise and which are significant. Indeed, we could have trained the model on the original 1920 × 1460 16-bit images without any background correction or removal, but doing this would make learning much slower and would require a considerable amount more training image data due to the added global context of the background. It would also make synthetic data generation more difficult because of the added complexity. By removing the global context, which varies with each image, the CNN is not required to learn it. It is also much easier to replicate the 8-bit images using the variational auto-encoder to generate synthetic data quickly. This was possible because the actual useful information was simple and was able to be easily extracted into a purer form. Synthetic data were used in the training of our CNN because capturing experimental data are expensive and the useful information in our experimental images is relatively straightforward and easily reconstructed in silico. By applying a background estimation filter to our images and subsequently generating a background-removed SBR image, the global context, which contains no useful information, is removed from the images, allowing for easy and fast synthetic data generation. An example of a synthetically generated image can be seen in Figure 2.

To ensure that synthetic data were representative of experimental data, visual inspection was used as well as numerical inspection. The reason visual inspection was used is because the 28 × 28-sized images can easily and quickly be inspected for major issues such as morphology density. Further, 50 sample images were chosen randomly and inspected for these two characteristics, while the average blob size was found numerically. The model was retrained until all randomly selected 50 images displayed similar morphologies and densities as the experimental images and their average sizes were found to be within 5% of the experimental images. As for the average pixel values of the blobs, it was decided that the total average values of all synthetically produced images should be within 10% of the average values of experimental images. It was not believed and subsequently found that the images must not exactly mimic those of experimental images in totality, only that the synthetic images closely resemble experimental images in size, morphology, density, and intensity. For this reason, both mask and target images were used in equal amounts for training of the VAE. To demonstrate that the generated synthetic data could be reliably used as CNN training data, the CNN model was trained using the 1000 synthetic data only. No experimental data were used for testing during this trial. The produced model was then used to predict 50 randomly chosen experimental data (which had not been used for training), and then the mean intersection-over-union was calculated for this process. It was found that the mean IOU for this procedure was 98.4, indicating that the synthetic data were not only effective for use as CNN training data but that the synthetic data could completely replace experimental data.

### 3.3. Model Training and Performance

**Figure 3:**
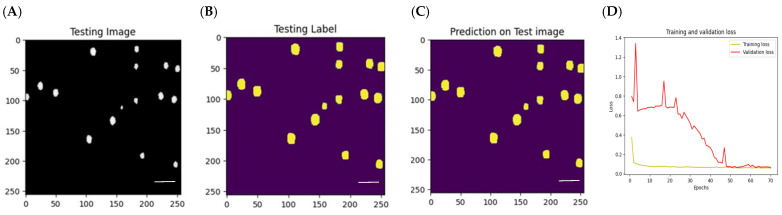
(**A**) Example of a 256 × 256 patched test image, originating from either a full-sized experimental or synthetic image; (**B**) Segmentation map generated by either ImageJ or AutoSEDIA; (**C**) Segmentation map predicted by CNN on image A; (**D**) Calculated Loss function. Scale bar: 200 nm.

50 full-sized 1920 × 1460 8-bit images were used for model evaluation purposes. These images included half experimental and half synthetically generated images. An average IOU was calculated for every complete 1920 × 1460 consisting of 48 256 × 256 patches each with its own mean IOU, then these results were averaged to give a single mean IOU for every 1920 × 1460 image. This global mean IOU was 98.9, indicating a high level of accuracy for this model’s prediction performance. Additionally, the loss function was calculated, and convergence was seen around 50 epochs. An example of the prediction results can be seen in Figure 3.

**Figure 4:**
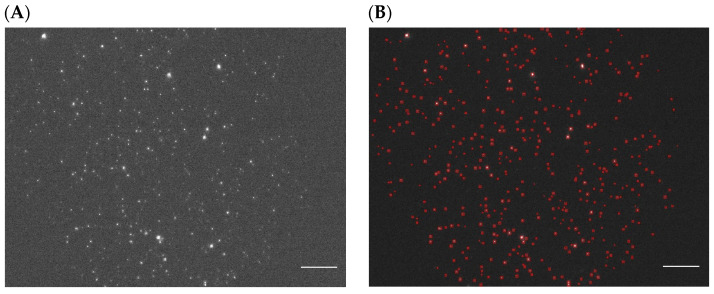

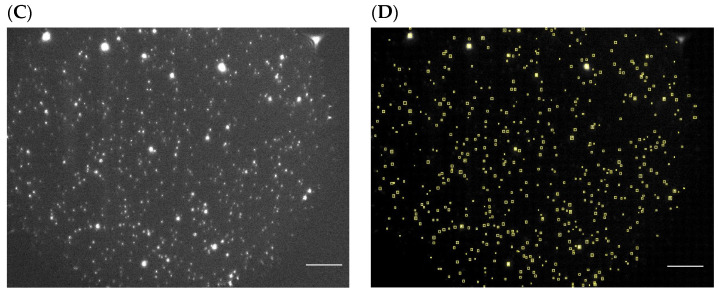
(**A**) Full-sized experimental fluorescence (mask) image; (**B**) Associated labeled mask image after segmentation map prediction and subsequent stitching; (**C**) Full-sized experimental dark-field (target) image; (**D**) Associated labeled target image after segmentation map prediction and subsequent stitching. Scale bar: 10 µm. Images were acquired using plasma EVs from a Stage III patient with EpCAM as the targeted surface marker.

Once the prediction was carried out on the smaller 256 × 256 patches, those patches were stitched back together to reform the original 1920 × 1460 image. This prediction procedure was used for all data that the model had not been trained on, i.e., all further new experimental data. An example of an experimentally gathered image set consisting of the fluorescence mask image and the corresponding dark-field target image where both the original images have undergone the entire prediction and stitching process can be seen in Figure 4.

### 3.4. EpCAM Profiling of Individual Plasma EVs from HER2-Positive Breast Cancer Patients at Different Stages

For simplicity, we chose HER2-positive breast cancer as the disease model to examine whether breast cancer could be detected at different stages. Using DISVT, our prior studies have demonstrated that exosomal HER2 can be used to detect HER2-positive breast cancer at Stage III as well as the early stage (stages I/II) by characterization of F_HER2_ from plasma EVs [24]. In this study, we used DISVT to examine whether different stages of HER2-positive breast cancer could be differentiated from other cancer markers with a pilot cohort of subjects (*n* = 7 for Stage I, *n* = 8 for Stage II, *n* = 9 for Stage III, *n* = 6 for Stage IV, and *n* = 10 for healthy donors).

**Figure 5:**
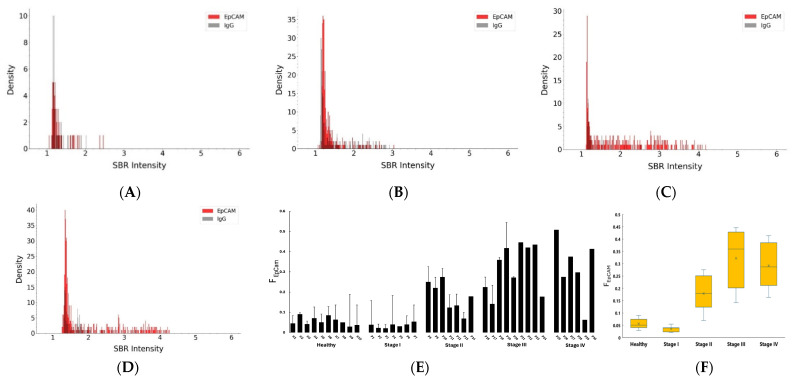
(**A**–**D**) The population density histogram of plasma EVs from a patient at each stage using EpCAM-conjugated AuNPs (red) and IgG-conjugated AuNPs (grey). (**A**) Stage I; (**B**) Stage II; (**C**) Stage III; (**D**) Stage IV; (**E**) Comparison of F_EpCAM_ of plasma EVs from different human subjects; (**F**) Box plot of F_EpCAM_ for patients and healthy donors.

Using DISVT in combination with the machine learning-assisted image analysis platform, we profiled EpCAM on individual plasma EVs from HER2-positive breast cancer patients and compared them with healthy donors. Our prior studies with a surface-enhanced Raman scattering (SERS)-based bulk method showed that exosomal EpCAM is a biomarker for the detection of HER2-positive breast cancer using plasma samples mainly from Stage III patients [47]. In this study, we further examine the diagnostic potential of EV’s EpCAM by measuring F_EpCAM_ of HER2-positive breast cancer at different stages. Figure 5A–D shows an example of the EpCAM profile on CD81-captured EVs for each patient group and Figure S1 for the healthy control using AuNPs bound with anti-EpCAM antibodies and AuNPs bound with IgG control. The F_EpCAM_ values for all subjects are shown in Figure 5E, and the box plot of these data are shown in Figure 5F. The F_EpCAM_ values for healthy donors and Stage I patients were negligible, with an average of 5.6% for healthy donors and 3.5% for Stage I patients. The average F_EpCAM_ for Stage II patients was 18% (range: 6%~28%), 32% (range: 14%~45%) for Stage III patients, and 29% (range: 16%~42%) for Stage IV patients. Statistical analysis with ANOVA showed that the F_EpCAM_ values of these patients were significantly different from that of the healthy control (*p* < 4.5 × 10^−^^3^) (Table S2). F_EpCAM_ values of the four stages of breast cancer patients were significantly different from each other (*p* < 0.04) except those between Stage III and Stage IV (*p* = 0.601). These results suggest that EpCAM on EVs can detect the HER2-positive breast cancer at Stage II, III, and IV, but not Stage I. It can differentiate the four stages except Stage III and Stage IV.

### 3.5. CD24 Profiling of Individual Plasma EVs from HER2-Positive Breast Cancer Patients at Different Stages

**Figure 6:**
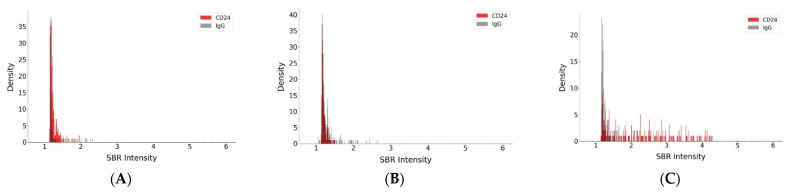

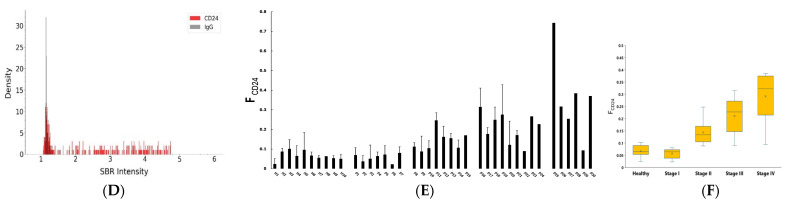
(**A**–**D**) The population density histogram of plasma EVs from a patient at each stage using CD24-conjugated AuNPs (red) and IgG-conjugated AuNPs (grey). (**A**) Stage I; (**B**) Stage II; (**C**) Stage III; (**D**) Stage IV; (**E**) Comparison of F_CD24_ of plasma EVs from different human subjects; (**F**) Box plot of F_CD24_ for patients and healthy donors.

Similar to the studies on EpCAM, we profiled CD24 on individual plasma EVs using DISVT and our machine learning-assisted image analysis platform, and the results are shown in Figure 6. The F_CD24_ values for healthy donors and Stage I patients were negligible, with an average of 6.7% for healthy donors and 5.7% for Stage I patients. The average F_CD24_ for Stage II patients was 14% (range: 8~25%), 21% (range: 8~32%) for Stage III patients, and 29% (range: 9~39%) for Stage IV patients. Statistical analysis with ANOVA showed that the F_CD24_ values of these patients were significantly different from that of the healthy control (*p* < 3.5 × 10^−^^3^) (Table S3). F_CD24_ values of the four stages of breast cancer patients were significantly different from each other (*p* < 0.05) except those between Stage III and Stage IV (*p* = 0.147). These results suggest that CD24 on EVs can detect HER2-positive breast cancer at Stages II, III, and IV, but not Stage I. It can differentiate the four stages except Stages III and IV.

## 4. Conclusions

Using machine learning, we were able to construct a fully automatic image analysis pipeline that is faster than our previous methods. Furthermore, this software can be used by other researchers who may be unfamiliar with programming or advanced image analysis techniques. This is important because, without this software, only advanced users can analyze data. Additionally, full automation allows for the possibility of commercialization. Without image analysis automation, commercialization is prohibitory, as non-automated analysis is impractical due to the advanced knowledge needed to conduct the analysis and the time required to perform it.

Utilizing DISVT with our machine learning-assisted image analysis platform and pilot human subjects, we demonstrate that EV’s CD24 and EpCAM can be used as biomarkers to detect breast cancer (HER2-positive) at Stages II, III, and IV. Both markers can differentiate between the four stages except Stages III and IV. These results suggest that CD24 and EpCAM can be used as auxiliary markers to help detect and differentiate HER2-positive breast cancer at different stages. In fact, EpCAM and CD24 are associated with HER2-positive breast cancer, which can be seen from their co-expression on cell lines (Figure S2). Thus, the three markers may be used as composite markers to detect and differentiate HER2-positive breast cancer at different stages.

Our image analysis software, along with our current results demonstrating that our newly developed diagnostic platform can target multiple cancerous markers and can differentiate cancer at specific stages, opens new opportunities for clinical applications. If our platform can become commercially viable, it would drastically assist in the effort to diagnose cancer early as well as monitor its progression. Monitoring cancer progression is essential for the correct administration of cancer therapies, as those therapies greatly depend on the stage of cancer. Furthermore, by using our platform in tandem with current cancer detection and monitoring methods, the monitoring process can be more tailored to specific patients. This is important because each patient will respond to the disease and therapies differently; therefore, the process of monitoring will be different for each patient.

It is noted that the feasibility of EV’s CD24 and EpCAM to detect breast cancer needs to be validated with a larger cohort and with other molecular subtypes (i.e., hormone-positive and triple-negative breast cancer). In addition, the DISVT methodology needs to be translated into an automatic methodology for use in a routine clinical setting. This can be achieved by integrating the procedures with the existing microarray technology for automated and high-throughput analysis.

## Data Availability

Data are contained within the article and Supplementary Materials.

## Supplementary Materials

The following supporting information can be downloaded at: https://www.mdpi.com/article/10.3390/nano14211739/s1, Table S1. The number of 1920 × 1460 full sized training images from either experimental or synthetically generated data and the number of patched created from useable data. Table S2. *p*-values for F_EpCAM_ between different groups of subjects. Table S3. *p*-values for F_CD24_ between different groups of subjects. Figure S1. (A) The population density histogram of plasma EVs from a healthy donor using EpCAM-conjugated AuNPs (red) and IgG-conjugated AuNPs (grey). (B) The population density histogram of plasma EVs from a healthy donor using CD24-conjugated AuNPs (red) and IgG-conjugated AuNPs (grey). Figure S2. Flow cytometry detection of HER2, CD24 and EpCAM on SKBR3 cells.
