# Supplementary material for: Single Vesicle Surface Protein Profiling and Machine Learning-Based Dual Image Analysis for Breast Cancer Detection
Source: Nanomaterials (Basel). 2024 Oct 30;14(21):1739. doi: 10.3390/nano14211739 (PMC11548014; doi:10.3390/nano14211739)
Supplement: Supplementary file 1 [file nanomaterials-14-01739-s001.zip › nanomaterials-3251688-supplementary.pdf]

Single Vesicle Surface Protein Profiling and Machine Learning-Based Dual Image Analysis for Breast Cancer Detection

Mitchell Lee Taylor 1,†, Madhusudhan Alle 1,† , Raymond Wilson Jr. 1, Alberto Rodriguez-Nieves 1 ,Mitchell A. Lutey 1 , William F. Slavney 1, Jacob Stewart 2, Hiyab Williams 2, Kristopher Amrhein 1,‡,Hongmei Zhang 3 , YongmeiWang 1 , Thang Ba Hoang 2 and Xiaohua Huang 1,*

1 Department of Chemistry, The University of Memphis, Memphis, TN 38152, USA

2 Department of Physics and Materials Science, The University of Memphis, Memphis, TN 38152, USA;

3 School of Public Health, The University of Memphis, Memphis, TN 38152, USA

* Correspondence: xhuang4@memphis.edu; Tel.: +1-901-678-1728

† These authors contributed equally to this work.

‡ Current address: Department of Chemistry and Physics, The University of Tennessee at Chattanooga, Chattanooga, TN 37403, USA.

Table S1. The number of 1920 x 1460 full sized training images from either experimental or synthetically generated data and the number of patched created from useable data.

Training Data	# of 1920 x 1460 Images	# of 256 x 256 Patched Images
Experimental Images	100	847
Synthetic Images	100	847

Table S2. p-values for F_{EpCAM} between different groups of subjects.

	Healthy	Stage I	Stage II	Stage III	Stage IV
Healthy	-	-	4.3×10^{-3}	1.4×10^{-4}	1.4×10^{-3}
Stage I		-	2.1×10^{-3}	8.6×10^{-5}	9.7×10^{-4}
Stage II			-	0.011	0.038
Stage III				-	0.601
Stage IV					-

Table S3. p-values for F_{CD24} between different groups of subjects.

	Healthy	Stage I	Stage II	Stage III	Stage IV
Healthy	-	-			
Stage I		-			
Stage II					
Stage III				-	0.147
Stage IV					-

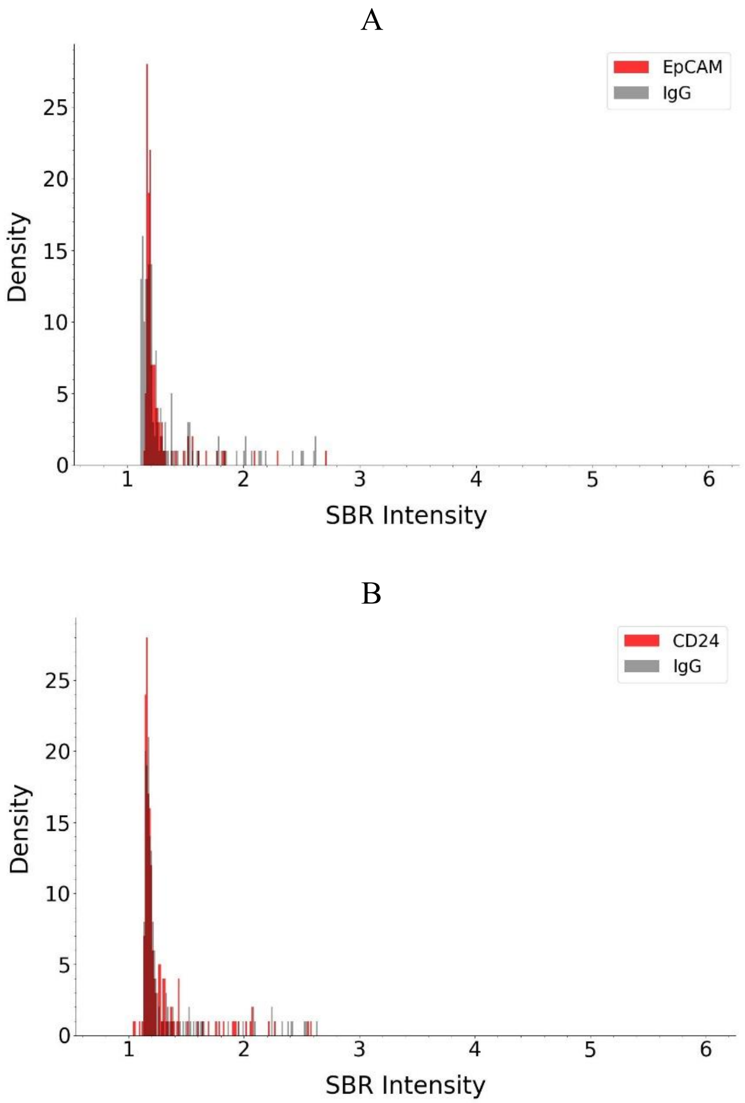

Figure S1. (A) The population density histogram of plasma EVs from a healthy donor using EpCAM-conjugated AuNPs (red) and IgG-conjugated AuNPs (grey). (B) The population density histogram of plasma EVs from a healthy donor using CD24-conjugated AuNPs (red) and IgG-conjugated AuNPs (grey).

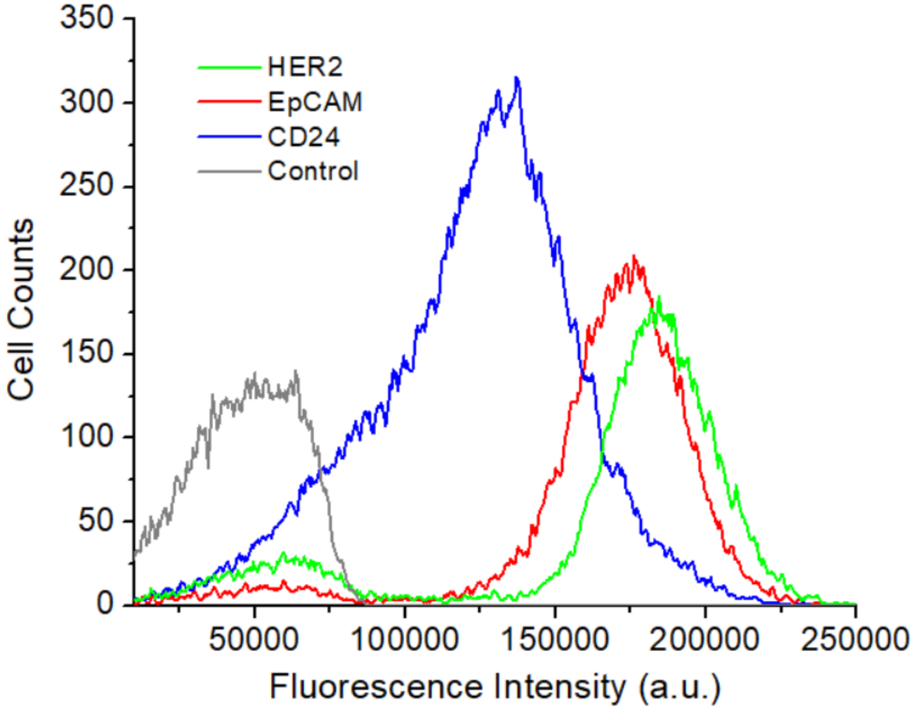

Figure S2. Flow cytometry detection of HER2, CD24 and EpCAM on SKBR3 cells.
